# Rapid Manufacturing Approach of an Ultrathin Moisture Sensor for Health Monitoring

**DOI:** 10.3390/s23094262

**Published:** 2023-04-25

**Authors:** Lan Zhang, Jian Lu, En Takashi, Sohei Matsumoto

**Affiliations:** 1 Device Technology Research Institute, National Institute of Advanced Industrial Science and Technology (AIST), Tsukuba 305-8564, Japan; 2 New-Generation Medical Treatment and Diagnosis Research Laboratory, National Institute of Advanced Industrial Science and Technology (AIST), Tsukuba 305-8566, Japan; 3 Faculty of Nursing, Nagano College of Nursing, Komagane 399-4117, Japan

**Keywords:** ultrathin moisture sensor, urine health monitoring, real-time measurement, rapid jet printing technology

## Abstract

This study developed a rapid manufacturing approach for a moisture sensor based on contactless jet printing technology. A compact measurement system with ultrathin and flexure sensor electrodes was fabricated. The proposed sensor system focuses on continuous urine measurement, which can provide timely information on subjects to ensure efficient diagnosis and treatment. The obtained results verify that the proposed sensor system can exhibit a typical responsivity of up to −7.76 mV/%RH in the high-sensitivity range of 50–80 %RH. A preliminary field experiment was conducted on a hairless rat, and the effectiveness of the proposed ultrathin moisture sensor was verified. This ultrathin sensor electrode can be fabricated in the micrometer range, and its application does not affect the comfort of the user. The ultrathin electrode sensors can be printed directly on the diaper or undergarment of the user for in situ urine health monitoring, particularly of infants and the elderly.

## 1. Introduction

The improved living standards and the development of modern society have rendered monitoring of health conditions a primary concern [1,2,3,4]. In particular, the real-time health monitoring requirements of the elderly and newborn infants have increased [5,6]. The measurement of physiochemical and biological parameters of pathological aspects can effectively reflect the health condition of the tested subject and provide a reference for doctors to execute early treatments [7]. Typically, the measurement of these parameters is based on three primary mediators: saliva, blood, and urine [8,9,10,11]. The collection of blood is an invasive sampling process and unsuitable for frequent use over a short period. Although saliva sampling is non-invasive, its applicability and accuracy have not been sufficiently investigated owing to the limited information available on saliva analytes. Therefore, continuous urine measurement is the simplest method for real-time health monitoring in terms of body fluids containing physical and chemical media. 

The major issue in real-time monitoring of urine is its effective collection, owing to the existing methods of urine collection being plagued by certain problems. Typically, the tested urine is collected in a special urine collection bag [12,13]. However, the users may experience discomfort owing to contact between the urine bag and skin and the leakage of the urine bag. Moreover, the urine collection bag does not comprise a real-time detection function. Therefore, sensing technology researchers have attempted to improve the efficiency of urine collection and reduce the discomfort of the user [14]. In the case of infants, a urine sensor can be placed on the diaper to continuously monitor the status of urine with minimal discomfort. In the case of the elderly, a urine volume test can be crucial because reasonable health monitoring can prevent the occurrence of fatal diseases [15,16]. 

**Figure 1:**
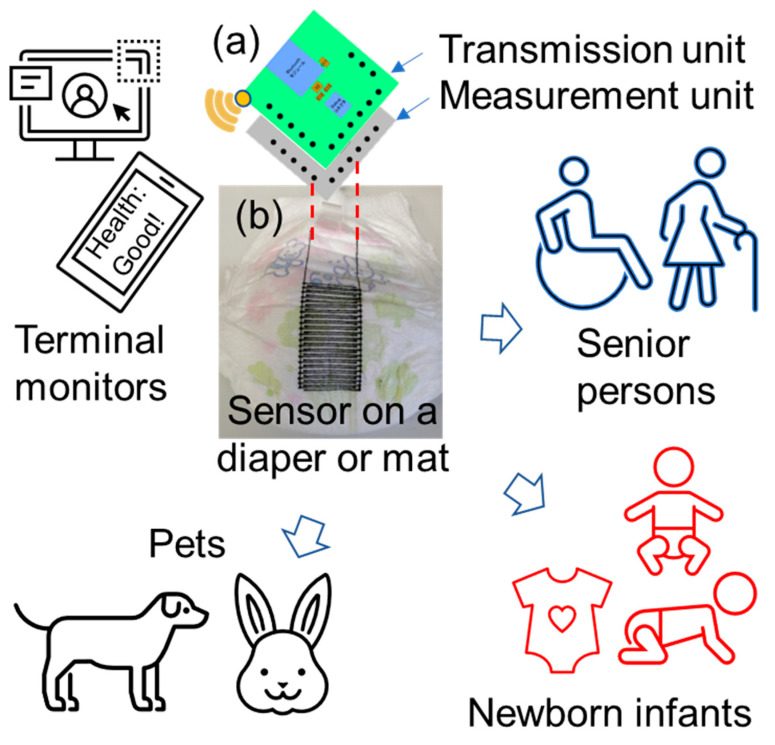
Conception and potential application of the ultrathin moisture sensor. (**a**) Schematic of the measurement and transmission boards in the sensor system. (**b**) A typical fabricated sensor on a baby diaper.

Therefore, this study developed an efficient fabrication method using moisture sensor electrodes for urine measurement based on rapid jet printing technology. The proposed moisture sensor system comprises testing electrodes and system circuit boards capable of measuring the urination status of the user. The proposed method can print the ultrathin sensor electrodes directly onto the diaper or undergarment of the user. The sensor electrode can be fabricated in the micrometer range and does not affect the comfort of the user. In fact, certain organizations have developed highly useful urine sensors that can be placed on undergarments or diapers for the detection of the urination status of the user in real time [17,18]. However, these sensors contain only one detection unit; thus, they are not that effective in detecting the distribution of urine. In contrast, the proposed moisture sensor fabrication technology exhibits good customization capabilities for different users. The proposed sensor electrodes can be printed as a single unit at the center of the diaper or distributed in an array at various defined locations on the diaper or undergarment. The performance of the given sensor was evaluated in terms of its physical and electrical characteristics. Moreover, a preliminary field experiment was conducted on a hairless rat, and the effectiveness of the proposed ultrathin urine sensor was verified. Figure 1 depicts the conception of the proposed sensor system and the potential application of the fabricated ultrathin moisture sensor. The fabrication platform was used to print the sensor electrodes directly onto the diaper. The electrodes were connected to the circuit boards via extension wires, and the circuit boards encompassed the measurement and transmission units. The users can monitor the status of the sensor in real time at terminal units via a Bluetooth signal. Moreover, the fabricated ultrathin moisture sensor is inexpensive and can be considered for several potential applications, such as time, volume, and distribution measurement of urine. In addition, the functions of the urine sensor system are expandable by changing the electrode materials and measurement circuit, which can facilitate the testing of multiple urine substances, such as various ions, proteins, urine occult blood, and other biological indicators. 

## 2. Manufacturing Platform of the Moisture Sensor Electrode

**Figure 2:**
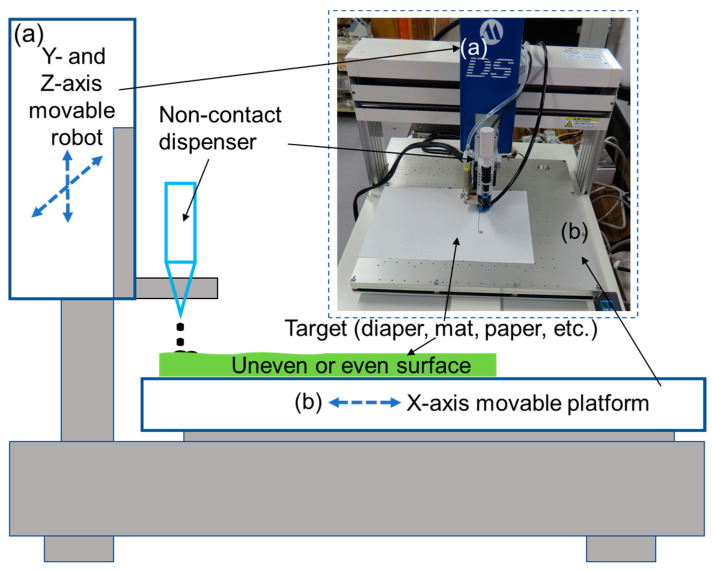
Rapid manufacturing platform of the moisture sensor electrode. (**a**) Y- and Z-axis movable robot. (**b**) X-axis movable platform.

Figure 2 shows the schematic of the rapid manufacturing system of the moisture sensor electrode; the inset in Figure 2 is a photograph of the manufacturing platform. The contactless print unit of the manufacturing platform was realized based on a jet dispenser. The jet dispenser was used to fabricate the sensor electrode, which can stably eject electrode material at a certain distance from the object being processed. The movable unit of the manufacturing platform was primarily composed of two parts: the robot arm that can realize Y- and Z-axis movement (Figure 2a) and the sliding table that can realize the X-axis movement (Figure 2b). The processed object can be set on the stainless-steel sliding table with a bearing area of 400 × 400 mm, which is sufficiently large to accommodate nearly all diapers, pet cushions, and mats. 

The proposed rapid manufacturing system exhibits several incomparable advantages over existing conventional manufacturing methods. The rapid manufacturing system is not a contact printing method. Further, the distance between the printhead of the jet equipment and processed material is adjustable up to 40 mm. This feature facilitates the production of device electrodes on uneven surfaces, which significantly expands the application scope of the proposed manufacturing system. In addition, owing to the proposed rapid manufacturing system using the jet method to fabricate the sensor electrodes, the electrode materials are replaceable by the simple changing of the electrode material container. This implies that the rapid manufacturing system is highly flexible in terms of fabricating different electrode materials, such as silver glue, conductive silver paste, and carbon resin. Finally, the sensor electrode pattern was designed employing special fluid-dispensing pattern editing software. Therefore, different electrode patterns can be manufactured by changing the design through the design software. This feature can significantly improve the flexibility of the equipment and satisfy the requirements of customers. Moreover, for different application fields, a polymer film with absorbent properties can be coated on the surface of the fabricated sensor electrodes to improve the sensitivity of the specific sensor system. 

## 3. Fabrication and Evaluation Methods

### 3.1. Laboratorial Fabrication and Evaluation Methods

A non-contact jet dispenser (AeroJet, Musashi Engineering Co., Ltd., Tokyo, Japan) was used to structure the sensor electrode, and suitable dispensing parameters were realized using a high-precision dispenser controller (MJET-4-CTR, Musashi Engineering Co., Ltd.). An air compressor (0.4LE-8SB, Hitachi Co., Ltd., Tokyo, Japan) was used to provide stable compressed air at 0.6 MPa to the dispenser. In addition, a high-precision frequent-use desktop robot (Shotmaster 400 DS, Musashi Engineering Co., Ltd.) was used to pattern the sensor electrodes. The layout of the electrode patterns was designed using fluid-dispensing pattern editing software (MuCAD III Ver. 1, Musashi Engineering Co., Ltd.). A bibulous paper (Crapo CM01S, Hisago Co., Nagoya, Japan) with a thickness of 0.13 mm was used as a print substrate in the evaluation experiment. A digital microscope (KH-7700 controller, MX-5040SZ lens, Hirox Co., Ltd., Tokyo, Japan) was used to observe the structure of the fabricated sensor electrode and evaluate the surface features of the fabricated vibration sensors. Furthermore, a constant climate cabinet (LHL-114, Espec Corporation, Osaka, Japan) was used to generate a setting ambient temperature and humidity environment to evaluate the fabricated sensors. Finally, an LCR meter (ZM2372, NF Corporation, Yokohama, Japan) was used to measure the capacitance and resistivity of the fabricated sensor electrodes.

### 3.2. Field Measurement Preparing and Monitoring Methods

**Figure 3:**
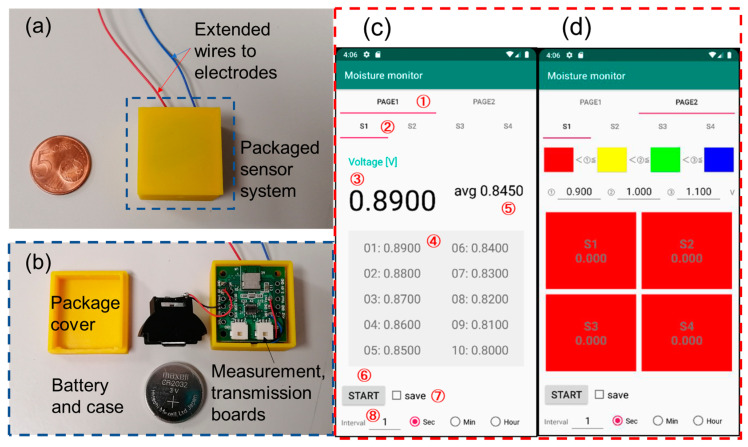
Packaged wireless sensor unit and user interface in the terminal of a smartphone. (**a**) Packaged sensor system. (**b**) Main components of the sensor system. (**c**) Monitoring page of the user interface. (**d**) Threshold setting page.

The final application of the proposed wireless moisture sensor was to be set on a diaper for measuring human urine. As the direct application to human beings is impractical, the proposed sensor should initially be tested on animals to verify its functional performance. A three-dimensional printer (Ultimaker 2, Ultimaker Co., Utrecht, The Netherlands) was used for fabricating the sensor module package. Figure 3a shows the casing of the sensing and transmission boards of the moisture sensor used for field measurements. The dimensions of the packaged sensor module were 24 mm × 24 mm × 12 mm. A CR2032 battery (Maxell, Ltd., Tokyo, Japan) was placed in the battery case to supply power to the sensor system (Figure 3b). For long-term and continuous monitoring applications, users can incorporate other batteries with larger capacities. Two signal-line wires were connected to the sensing board with the sensor electrodes. During field measurement, the sensor electrodes were placed on the back of the mat or on the interlayer, where the sensor was used to test the change in capacitance.

A smartphone (Pixel 3a, Google Co., Mountain View, CA, USA) was used to monitor the moisture sensor output value via Bluetooth transmission. An application program primarily comprising moisture measurement and Bluetooth transmission was developed successfully. The Bluetooth module Raytac MDBT42V-P512KV2 was used to realize the wireless transmission function under the communication specifications of V5.0 Bluetooth low energy. A customized Android application with a friendly user interface was coded in the C and Kotlin languages. Figure 3c,d show the user interface of the moisture sensor system with the smartphone being used as the terminal device. Figure 3c shows the monitoring page of the moisture sensor system. The measurement interval time could be adjusted from 1 s to 10 h. The latest 10 data points could be directly observed on the screen, and all the data were saved in the memory. Figure 3d shows the threshold setting page of the moisture sensor system. Four monitoring ranges could be set in different colors to facilitate the identification of the testing conditions by the users at a glance. For practical application in future, the proposed sensor can be calibrated by users through the output voltage and moisture value formula via the next software upgrade. By converting the voltage output of the sensor, users can directly obtain the measured moisture value.

## 4. Measurement Results

### 4.1. Comparison of the Electrode Fabrication Materials

A non-contact jet dispenser was implemented in this study for fabricating the sensor electrodes. The dispenser nozzle can eject the specified material with a certain liquid viscosity in the range of 50–300,000 mPa⋅s. Conductive silver paste and carbon resin were selected as the materials for electrodes, considering their safety, stability, and reasonable electrical conductivity. Following the patterning of the two materials on the object, post-bake treatment processes of both materials were measured and compared. The results indicate that the silver paste should be baked in the oven at 80 °C for a minimum of 30 min, whereas the carbon resin can be adequately cured at a room temperature of 25 °C. We measured the internal resistance of the cured sensor electrodes using different materials with the typical values of 14.9 mΩ·cm and 11.5 Ω·cm for silver paste and carbon resin, respectively. As silver-paste-based sensor electrodes exhibit better ductility and internal resistance, they are more suitable for harsh environments and research fields. However, the price of carbon resin is less than one-fifth that of the silver paste. Because the production cost directly affects the commercialization of the proposed technique, carbon-resin sensors may be advantageous in terms of its promotion in large-scale empirical experiments and commercialization processes, owing to their low cost. However, high internal resistance inevitably decreases the output responsivity and increases the manufacturing complexity of measurement circuits, which affects the practical application of carbon-resin-based sensor electrodes. 

### 4.2. Characteristics of the Fabricated Sensor Electrodes

**Figure 4:**
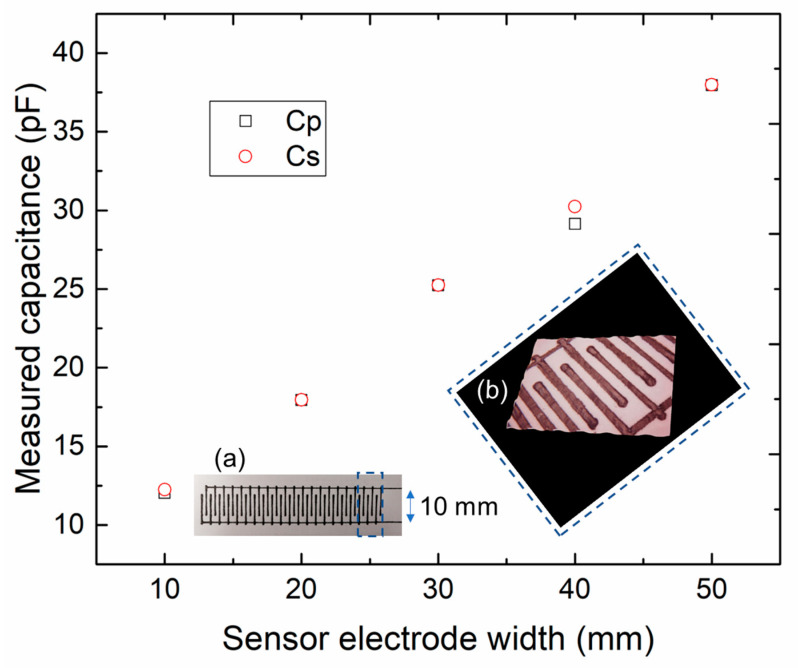
Measured capacitance of the sensor electrodes with different comb widths. (**a**) Optical image of the typical sensor electrode with a comb width of 10 mm. (**b**) Magnified optical image of the electrode structure.

Five types of sensor electrodes with widths in the range of 10–50 mm were fabricated using the rapid manufacturing system. The capacitances in parallel (Cp) and series (Cs) were comprehensively measured using the LCR meter. Figure 4 shows the measured capacitance of the typical sensor electrodes with different comb widths. Figure 4a illustrates the optical image of a typical sensor with a 10-mm-wide comb structure, and Figure 4b shows the magnified optical image of the sensing electrode structure. A 10-mm-wide comb sensor has a total length of 100 mm, with the sensing part of the comb measuring 60 mm in length. Further, the sensor electrode structure has an average thickness of 0.25 mm. The experiment was performed at a stable ambient temperature of 25 °C. The measured Cp values of the 10-, 30-, and 50-mm-wide electrodes were 12.04, 25.23, and 37.95 pF, respectively, and the electrode capacitance of the sensor increased with the increase in the width. The adequate linearity between the sensor electrode width and measured capacitance can provide an appropriate reference for designing the sensor in different applications. The optical image (Figure 4a) indicates that the comb-shaped sensor electrode can be successfully printed on the substrate. The two comb-shaped sensor electrodes were well-fabricated and maintained a space of approximately 1 mm. Moreover, the electrode structure of the sensor exhibited a reasonably smooth surface and uniform width, as observed in the magnified optical image (Figure 4b). Due to the inherent characteristics of the manufacturing process, the fabricated sensor electrodes were expected to suffer from some level of manufacturing deviation, which could be controlled within a reasonable range. To ensure consistency in subsequent experiments, we used the silver paste sensor electrode for characteristic evaluation. 

### 4.3. Evaluation of the Proposed Sensor Electrode

**Figure 5:**
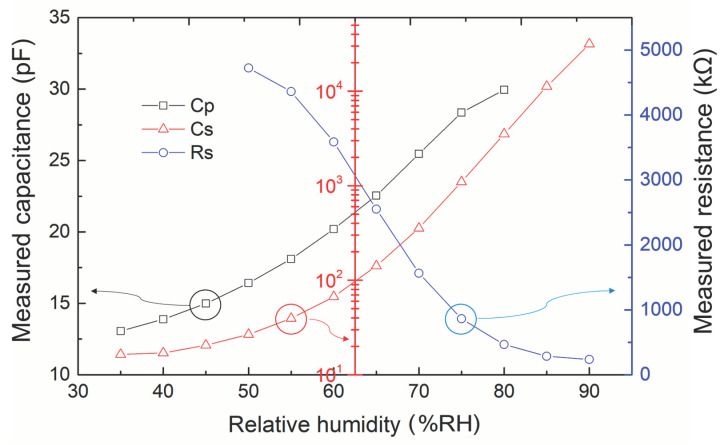
Comparison of the measured Cp, Cs, and Rs values in terms of varying relative humidity.

The values of Cp, Cs, and series resistance (Rs) of a typical fabricated sensor under different testing environments were comprehensively measured. The fabricated sensor electrodes were placed in the test cabinet with a well-programed environment of controlled humidity and temperature. The humidity change was measured in the range of 35–90 %RH under a constant temperature of 30 °C. The LCR meter was placed outside the cabinet chamber, and an extension wire cable was connected to the electrode for recording the output data. Figure 5 presents a comparison of the measured Cp, Cs, and Rs values, varying in terms of the relative humidity of the environment. The Cp and Cs values of the sensor electrode were measured was 13.06 and 16.40 pF, respectively, at identical conditions of 35 %RH. The Cp value of the proposed electrode increased with increase in the humidity, and the measured Cp values increased from 16.42 to 29.95 pF in the testing range from 50–80 %RH. Similar to the Cp measurement results, the measured Cs values changed proportionally with increasing humidity conditions. 

**Figure 6:**
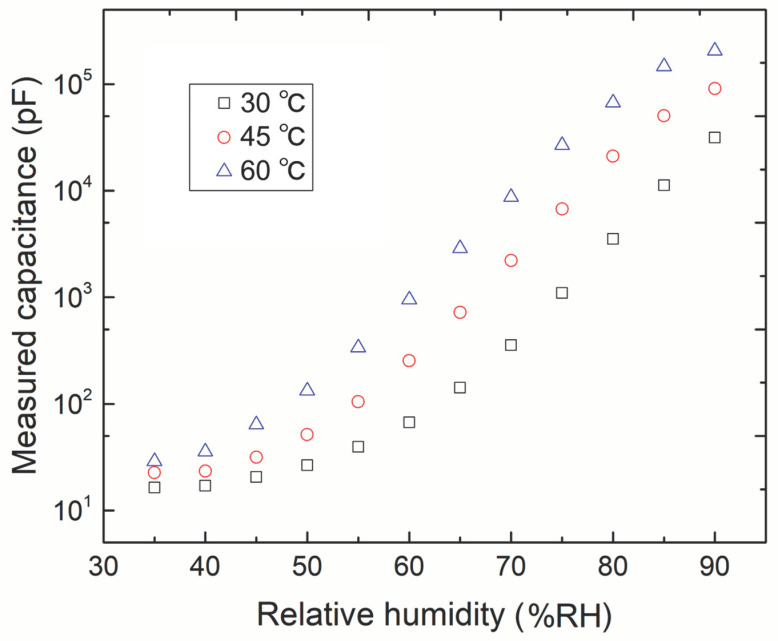
Comparison of the measured Cs values under different temperatures in terms of relative humidity.

A correlation was determined between relative humidity and environmental temperature. Therefore, the following experiments were conducted to verify the capability of the sensor to measure relative humidity across a wide range of temperatures. Figure 6 presents a comparison of the measured Cs values with different temperatures in terms of relative humidity. The humidity changed in the range of 35–90 %RH under three temperatures of 30, 45, and 60 °C. The following are the implications of the measurement results in Figure 6. (1) The results can be used as reference data for the subsequent work of sensor calibration, and (2) the measurement results confirmed that the electrodes of the proposed sensor exhibited a relatively high responsivity in the high humidity range; the second feature satisfies the usage requirements of moisture monitoring for diapers or mats. 

### 4.4. Development of Measurement System Circuits and Evaluation Results 

We completed the abovementioned evaluation experiments using high-precision testing instruments. Nevertheless, simplifying the measurement circuits and the signal processing system to a compact and wearable size is essential for realizing the practical application of the proposed sensor system. Therefore, this study adopted a simple circuit to detect the capacitance value between the sensor electrodes. A monolithic timing circuit was used to generate a square wave signal, which became a circular integral wave after passing through the integral circuit comprising the coefficient resistance and capacitance of the sensor electrode. The sensor electrodes were placed in the testing environment, and the capacitance changes between the electrodes were correlated with the humidity changes. The wave height of the integral waveform decreased when the composition of the capacitance of the sensor electrode increased. Finally, the output voltage decreased when the humidity on the sensor electrodes was higher. 

**Figure 7:**
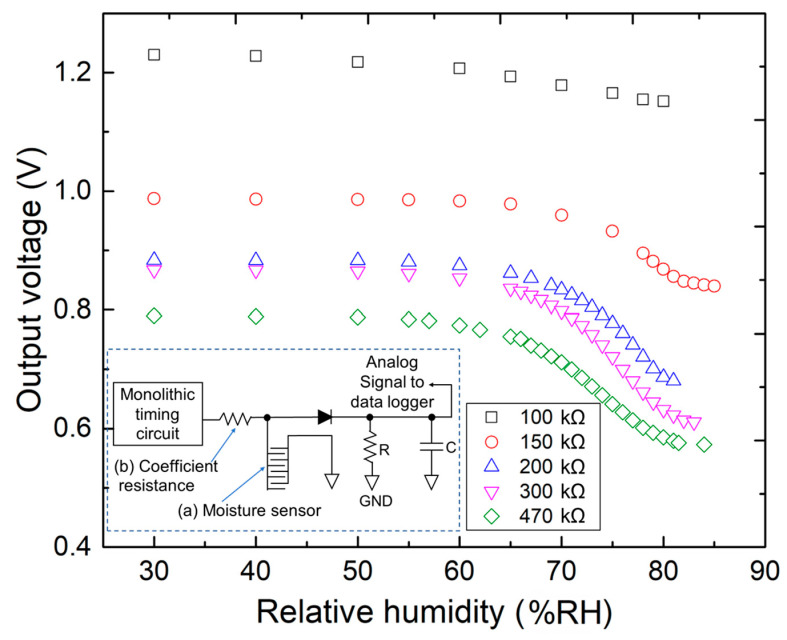
Comparison of the output voltage considering the relative humidity with different coefficient resistance values. (**a**) Sensor electrodes in the measurement circuit. (**b**) Coefficient resistance resistor in the measurement circuit.

Figure 7 presents a comparison of the output voltage with different coefficient resistance values while considering the relative humidity. The inset of Figure 7 shows the measurement circuit diagram, wherein the proposed electrode of the moisture sensor (Figure 7, inset (a)) is connected to the monolithic timing circuit. Moreover, a coefficient resistor is connected between the sensor electrode and the monolithic timing circuit in the integral circuit; this is necessary for adjusting the initial offset voltage and determining the responsivity of the sensor (Figure 7, inset (b)). Therefore, several coefficient resistors with alternative resistance were set in the measurement circuit. The high-precision electrical resistors were set in the measurement circuit, with resistance values in the range of 100–470 kΩ. The performance of the measurement circuit was comprehensively evaluated, and the optimal resistance value of the given resistor was determined. The analysis results (Figure 7) indicate that the measurement circuit was set with a typical coefficient resistance of 100 kΩ, and average responsivities of −1.57 mV/%RH and −2.22 mV/%RH were observed in the ranges of 30–80 and 50–80 %RH, respectively. Conversely, the measurement circuit with a coefficient resistance of 300 kΩ exhibited the highest responsivity, with –4.70 mV/%RH in the range of 30–80 and −7.76 mV/%RH in the high-sensitivity range of 50–80 %RH. Therefore, the coefficient resistance of 300 kΩ was packaged in the sensor system for subsequent experiments.

### 4.5. Experiment Results of Field Measurement

**Figure 8:**
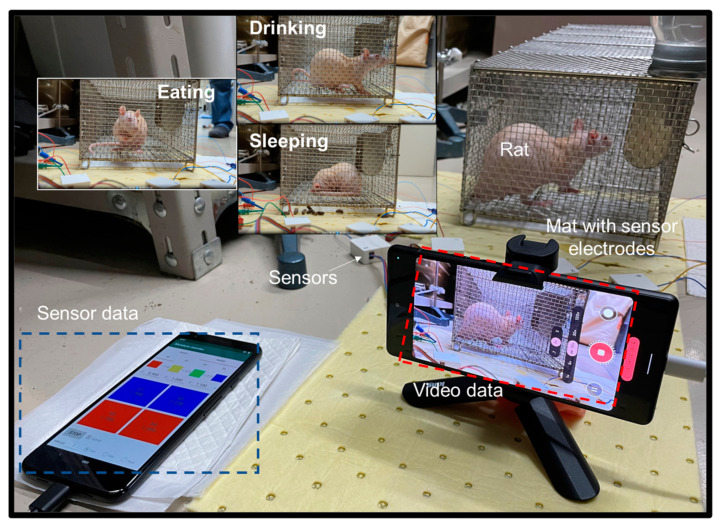
Optical image of the hairless rat and moisture sensor system in the field measurement. The insets show images of rats in different states.

Figure 8 shows the optical image of the rat and moisture sensor system in the field measurement. The inset images show the rats in different states. The operation process of this empirical experiment was conducted as follows. An eight-week-old healthy male hairless rat (HWY/Slc, Japan SLC, Inc. Hamamatsu-shi, Japan) was used in this study. The rat was placed in a standard cage (240 × 160 × 160 mm in size). The rat was fed a standard chow diet and provided free access to water. Moreover, no changes were made to its feeding lifestyle during the experiment. That is, food, water, and light were provided and adjusted as usual; in addition, the rat was not contacted or disturbed during the experiment. The mat was designed to fit the rat cage and was trimmed to the size of 240 × 160 mm. Two sensor electrodes of the same size were set on the mat to test the amount of excretion in different locations. The field measurement was conducted for 6 h. The data sampling interval was set to 1 s. In addition, the ambient temperature in the animal room was 25 °C, and the relative humidity was 50%.

**Figure 9:**
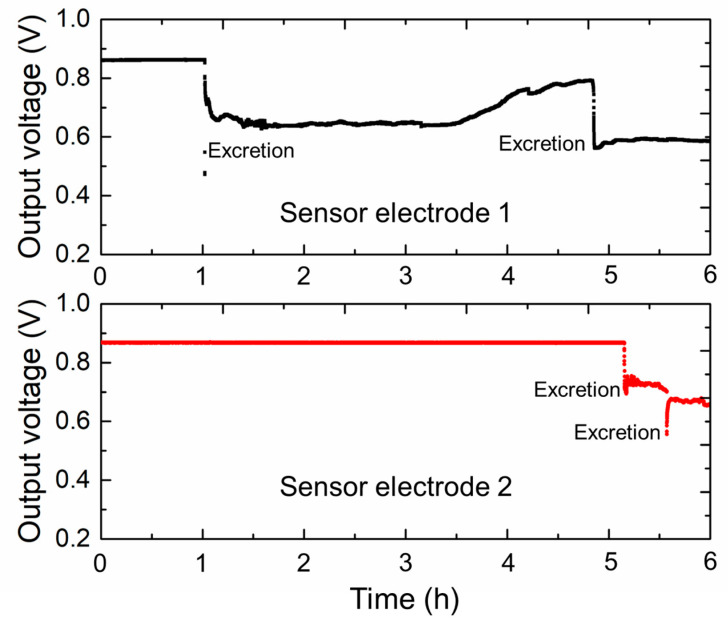
Voltage output of moisture sensor electrodes in field measurement.

Figure 9 shows the measurement results of the moisture sensor with two electrodes in field measurement. The two sensor electrodes 1 and 2 were set on the mat, which measured 150 mm in length with a center-to-center distance of 46 mm. The mat with sensor electrodes was placed 20 mm below the metal cage, thus facilitating the accurate measurement of excretion by the rat while preventing any interference from the metal cage or the movements of the rat. We observed that the two sensor electrodes on the mat initially yielded a relatively consistent output (approximately 0.86 V), and no special initialization operation was required to remove the offset. One hour into the experiment, sensor electrode 1 produced an induction signal, whereas sensor electrode 2 produced no response output. Therefore, we can assume that the rat excreted relatively far away from sensor electrode 2. Around the fifth hour of the experiment, the new induction signals were recorded at sensor electrodes 1 and 2, indicating that the excretion occurred in the central area of the mat. 

The preliminary field measurement results demonstrated that the developed moisture sensor could measure the mat condition of the rat in real time and maintain a stable baseline for the standby process. For field experiment preparation, a long-term stability experiment can be considered and implemented comprehensively. We tested the long-term stability of a representative sensor over four weeks at a frequency of one test per week. The sensor always maintained a stable initial output voltage value. As the interval time between diaper or mat changes is usually not particularly long, the continuous long-term experimental time range for the proposed sensor electrodes can be reduced to a few hours. The output results of electrode 2 also demonstrated that the sensor system exhibited reasonably continuous output stability within 5 h. 

## 5. Discussion and Outlook

Due to limitations in the sensor electrodes’ patterning and the number of tests, the preliminary results of the single animal experiment cannot be used as a quantitative standard for analyzing rat excretion. However, the qualitative results of the preliminary animal experiment can provide a framework for future improvement. First, the sensor electrode pattern could be modified to enable the monitoring of the full area of the mat. Second, increasing the number of channels to 4, 16, or higher can improve the spatial resolution of the sensor system. With optimized sensor system construction and comprehensive experiments, researchers can obtain holistic statistical data for urine monitoring research. Additionally, related studies in this field are ongoing. The camera recordings can be used to analyze the effect of urination and defecation of the target rat on the sensor output, and the relationship between sensor output and rat action can be discussed in the future. In subsequent studies, we intend to report on the investigations and results of animal experiments and field measurement analyses. 

## 6. Conclusions

This study developed a novel moisture sensor system with extended sensing electrodes to perform urination status measurements. The rapid jet printing technology enabled ultrathin sensor electrodes to be printed directly onto the diaper or undergarment of the user. The ultrathin sensor electrode was fabricated in the micrometer range and did not significantly affect the comfort of the user. Further, the proposed sensor system exhibited a responsivity of up to −7.76 mV/%RH in the high-sensitivity range of 50–80 %RH. A preliminary field experiment was conducted on a hairless rat, and the effectiveness of the proposed ultrathin moisture sensor was verified. Thus, once calibrated, the sensor can be utilized in various application fields related to urine detection.

## Data Availability

All data are true and reliable.
